# Plant Dependence on Rhizobia for Nitrogen Influences Induced Plant Defenses and Herbivore Performance

**DOI:** 10.3390/ijms15011466

**Published:** 2014-01-21

**Authors:** Jennifer M. Dean, Mark C. Mescher, Consuelo M. De Moraes

**Affiliations:** 1 Center for Chemical Ecology, Department of Entomology, Pennsylvania State University, 501 Agricultural and Life Sciences Building, University Park, PA 16802, USA; E-Mails: jmdean7@gmail.com (J.M.D.); mescher@usys.ethz.ch (M.C.M.); 2 Department of Environmental Systems Science, ETH Zürich, Zürich 8092, Switzerland

**Keywords:** *Glycine max*, *Bradyrhizobium japonicum*, *Helicoverpa zea*, *Aphis glycines*, jasmonic acid, rhizobia, herbivory

## Abstract

Symbiotic rhizobia induce many changes in legumes that could affect aboveground interactions with herbivores. We explored how changing the intensity of *Bradyrhizobium japonicum*, as modulated by soil nitrogen (N) levels, influenced the interaction between soybean (*Glycine max*) and herbivores of different feeding guilds. When we employed a range of fertilizer applications to manipulate soil N, plants primarily dependent on rhizobia for N exhibited increased root nodulation and higher levels of foliar ureides than plants given N fertilizer; yet all treatments maintained similar total N levels. Soybean podworm (*Helicoverpa zea*) larvae grew best on plants with the highest levels of rhizobia but, somewhat surprisingly, preferred to feed on high-N-fertilized plants when given a choice. Induction of the defense signaling compound jasmonic acid (JA) by *H. zea* feeding damage was highest in plants primarily dependent on rhizobia. Differences in rhizobial dependency on soybean did not appear to affect interactions with the phloem-feeding soybean aphid (*Aphis glycines*). Overall, our results suggest that rhizobia association can affect plant nutritional quality and the induction of defense signaling pathways and that these effects may influence herbivore feeding preferences and performance—though such effects may vary considerably for different classes of herbivores.

## 1. Introduction

Soil-dwelling microbial plant symbionts such as mycorrhizal fungi and nitrogen-fixing bacteria (rhizobia) can have profound impacts on plant ecology via influences on soil fertility, plant growth, and interactions between plants and other organisms [1–5]. Plant antagonists are often sensitive to changes in plant phenotypes mediated by symbionts, as demonstrated by the diverse effects of mycorrhizal fungi on plant-herbivore interactions [1,6–9]. There is evidence that rhizobia can similarly influence plant interactions with insect herbivores [3,10–12], but—apart from the well-documented effects of rhizobia presence/absence on total nitrogen levels and consequent effects on herbivores—the mechanisms underlying rhizobia-mediated changes in plant resistance to herbivores are poorly understood.

The co-evolved relationship between legumes and rhizobia is characterized by the supply of fixed nitrogen from the microbial partner to the plant in exchange for plant-produced carbon resources and a protective root nodule within which the bacteria live [13]. Underlying changes in host plant signaling and nutritional quality due to this association with rhizobia are likely to influence herbivores. The initial reaction of host plants to colonization by rhizobia resembles the response to pathogens, yet this stimulation of the plant immune system is subsequently suppressed as symbiotic signaling is initiated [14]. As the interaction continues, legume plants exert a significant degree of control over their facultative mutualism with rhizobia, modulating the intensity of the association in response to several factors, particularly soil nitrogen levels (as discussed below). Plant suppression of nodulation appears to be activated by signaling molecules from the shoots, likely including the phytohormones salicylic acid (SA) and jasmonic acid (JA), which have previously been implicated in induced plant defenses against herbivores [15–19].

Interactions between signaling pathways activated by rhizobia and those mediating induced defense responses may be expected to have significant impacts on plant-herbivore interactions. In response to attack by chewing herbivores, JA accumulates within plants and initiates a cascade of defense-related events, leading to the production of defensive compounds (e.g., toxins or digestibility inhibitors) and the emission of induced plant volatiles, which serve as foraging cues for herbivores’ natural enemies [15–18]. The SA-mediated signaling pathway is most often associated with plant defenses against pathogens [19,20] and abiotic stress [21], although SA is also induced by (and mediates effective defenses against) piercing-sucking herbivores [22] and parasitic plants [23]. Previous studies have demonstrated mutual inhibition between the SA- and JA-mediated signaling pathways and shown that crosstalk resulting from simultaneous interactions with multiple plant antagonists compromise induced defense responses against insect herbivores [24–26]. To the extent that rhizobia induce, or influence, the induction of these or other phytohormone signaling pathways, the intensity of rhizobia association might be expected to influence induced plant defenses against insect herbivores.

Rhizobia association also influences the nutritional quality of host plants, with the forms of nitrogen transported from the roots to the shoots of inoculated plants being quite different from those of plants receiving nitrogen fertilizers. In association with rhizobia, legumes originating from temperate regions transport nitrogen as the amides glutamine or aspargine, while tropical legumes—including soybean (*Glycine max* L.)—transport nitrogen as the ureides allatoin or allantoic acid [27]. Without rhizobia, nitrogen is typically transported to the shoots and assimilated into leaves as inorganic nitrate or ammonium [27]. As levels of available nitrogen in the soil increase, plants’ dependency on rhizobia for nitrogen decreases [28], and plants reduce the intensity of association with rhizobia. Consequently, the addition of nitrate to rhizobia-inoculated soybean plants reduces the number of functional rhizobia nodules and alters the relative prevalence of different nitrogen forms in leaves—decreasing ureides (e.g., allantoin and allantoic acid) and increasing nitrate in a dose-dependent manner, while maintaining equivalent total N levels [29,30]. Nitrogen is a key nutrient for herbivores, and the form(s) of nitrogen available can affect herbivore fitness and feeding behavior [31–33]. Differences in the quality of foliar nitrogen between rhizobia-inoculated and non-inoculated plants, such as the ratio of ureide nitrogen to nitrate nitrogen, are thus likely to have effects on herbivore feeding efficiency [33–35].

The purpose of the current study was to explore how the intensity of the rhizobial interaction influences aboveground plant-herbivore interactions. By growing rhizobia-inoculated soybean under a range of soil nitrogen levels established though fertilizer treatments, we were able to manipulate plants’ dependency on rhizobia to meet their N needs. As expected, this manipulation led to variation in the intensity of the rhizobia association and dose-dependent changes (as described above) in associated plant traits (e.g., the forms of nitrogen stored in leaves) even as total plant nitrogen levels remained similar. We then examined how this range of dependency on rhizobia affected: (i) feeding by the chewing, leaf-feeding soybean podworm (*Helicoverpa zea* Boddie; Lepidoptera) and the phloem-feeding soybean aphid (*Aphis glycines* Matsumura; Hemiptera); and (ii) herbivore-induced accumulation of the defense signaling compounds JA and SA.

## 2. Results and Discussion

### 2.1. Effects of Nitrogen Source on Plant Characteristics

**Figure 1.**
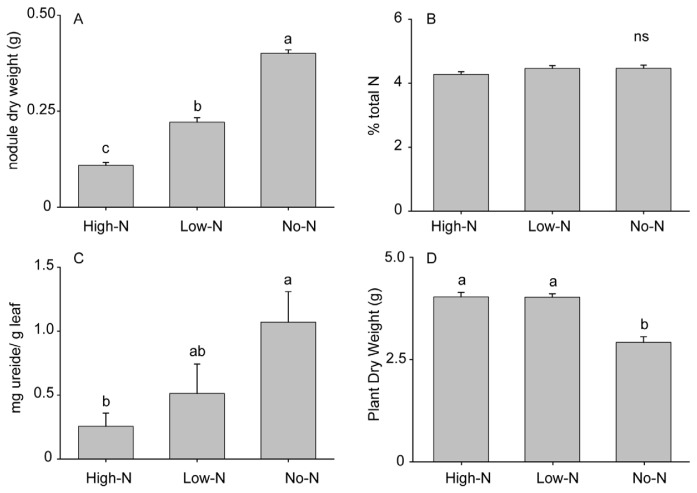
Characteristics of 5-week old plants inoculated with rhizobia, but given different levels of nitrogen fertilizer: (**A**) nodule dry weight; (**B**) percent total nitrogen; (**C**) ureide concentration (as allantoin) in the leaves; (**D**) aboveground plant dry mass. High-N = High N fertilizer treatment, Low-N = Low N fertilizer treatment, and No-N = no fertilizer. Different lowercase letters indicate significant differences for that characteristic, while n.s. indicates no significant difference. Results shown as means ± S.E.

We examined the effects of variation in the intensity of the plant-rhizobia association by manipulating levels of available soil N, resulting in variation in nodulation intensity and soybean plants’ dependence on rhizobia-derived N. As demonstrated in previous studies [29,30], available soil N under our growing conditions inhibited rhizobial colonization of the soybean roots in a dose-dependent manner. Plants receiving no supplemental nitrogen (No-N) were primarily dependent on rhizobia, as reflected by intensely nodulated roots, while plants receiving the highest nitrogen concentrations (High-N) had very few nodules (Figure 1A; *F*_2,15_ = 225.3; *p* < 0.001). Regardless of soil N availability, plants maintained similar total N levels (Figure 1B; *F*_2,27_ = 1.52; *p* = 0.236); however, the concentration of ureides in the leaves decreased with increasing N fertilizer (Figure 1C; *F*_2,12_ = 4.30; *p* = 0.039). Plants fertilized with low amounts of nitrogen (Low-N) exhibited an intermediate level of rhizobial dependence and nodulation. Complete dependency on rhizobia resulted in smaller plants, as No-N plants produced less aboveground and below-ground biomass than High-N and Low-N plants (Figure 1D; *F*_27,29_ = 33.6; *p* < 0.001).

Under low-nitrogen conditions, legumes derive significant fitness benefits from associating with rhizobia [36]. As soil N levels increase, the costs of cooperation can outweigh the cost of direct N acquisition, resulting in a plant-controlled decrease in nodulation and consequently in changes in plant traits influenced by the rhizobial interaction [29,37,38]. Soybean plants maintained a consistent level of total foliar nitrogen across each of our treatments, demonstrating their ability to modulate rhizobia associations to meet overall N needs. As we increased the concentration of soil N, the mass of root nodules, which enclose the nitrogen-fixing bacteroids, was significantly reduced. Complete dependency on rhizobia for N needs resulted in smaller plants, possibly reflecting the cost incurred by plants in supplying carbon resources to the microbial partner. As expected, the proportion of N incorporated into leaves in the form of ureides was also reduced as soil N increased and rhizobial dependence decreased.

### 2.2. Soybean-Rhizobia Interactions with a Chewing Herbivore

#### 2.2.1. Growth Rate and Preference Tests

Growth of the soybean podworm was affected by the rhizobial dependency of soybean. Podworm larvae confined to cups containing similarly-aged detached leaves from the different treatments had higher relative growth rates when fed leaves from No-N plants than leaves from High-N plants (*F*_2,58_ = 3.27; *p* = 0.0454; Means ± SE: No-N = 0.881 ± 0.059 g/g/day, Low-N = 0.740 ± 0.061 g/g/day, High-N = 0.677 ± 0.051 g/g/day). The total amount of leaf area consumed by larvae was similar across nutrient treatments in the detached leaf trials (*F*_2,51_ = 0.98; *p* = 0.3839; Means ± SE: No-N = 664.3 ± 50.5 mm^2^, Low-N = 555.3 ± 53.2 mm^2^, High-N = 611.2 ± 58.7 mm^2^).

**Figure 2.**
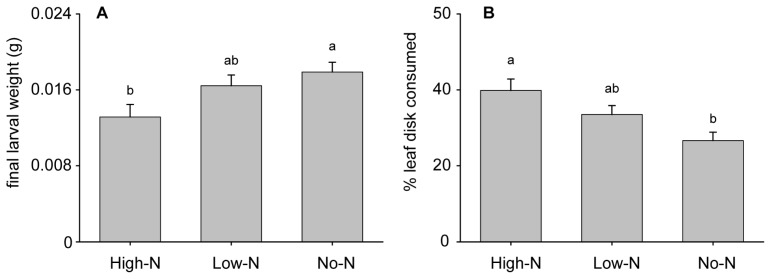
Effects of dependence on rhizobial N by 5-week old soybeans on (**A**) growth of *H. zea* larvae in a no-choice assay (starting as neonates), and (**B**) larval feeding preference in a 3-way choice test (third instar larvae). High-N = High N fertilizer treatment, Low-N = Low N fertilizer treatment, and No-N = no fertilizer. Different lowercase letters indicate significant differences. Results shown as means ± S.E (*n* = 30).

Further growth rate tests, in which larvae, starting as neonates, were monitored for eight days on intact plants, confirmed this trend of greater larval growth on plants receiving no nitrogen fertilizer (and thus primarily dependent on rhizobia) than on High-N plants (Figure 2A; *F*_2,50_ = 3.95; *p* = 0.026). In both bioassays, larvae feeding on Low-N plants/leaves grew an intermediate amount between those fed High-N and No-N plants/leaves.

The ability of podworm larvae to discriminate between leaves of soybeans receiving different nitrogen treatments was assessed through three-way choice assays. While the larvae took bites from each type of leaf, there was a distinct preference for High-N over No-N leaves (Figure 2B; *F*_2,89_ = 6.67; *p* = 0.002), despite the superior performance on No-N leaves reported above.

While total plant N levels were similar across the different fertilizer treatments, changes in the composition of N forms are likely to influence herbivore feeding. As plant dependency on rhizobia increased, so did the concentration of ureides, the transported nitrogen forms associated with rhizobia on soybeans. The ability of insects to efficiently metabolize nitrogenous compounds such as ureides remains unclear. Wilson and Stinner [35] observed decreased podworm pupal weights and longer development times when the ureide allantoin was added to artificial diets, and concluded that the larvae cannot efficiently utilize ureides as a N source. However, our experiments—no-choice tests with detached leaves and whole plants—revealed that podworm larvae grew best on plants primarily dependent on rhizobia for N (the No-N treatment) and thus having the highest levels of ureides. The apparent discrepancy between these findings may stem from differences in feeding studies conducted with artificial diet *versus* actual leaves, as the former may fail to include particular phytocompounds needed for proper utilization of organic N compounds. For example, degradation enzymes found in legume foliage [39] might be used by caterpillars to metabolize ingested ureides.

#### 2.2.2. Induced Hormone Accumulation

**Figure 3.**
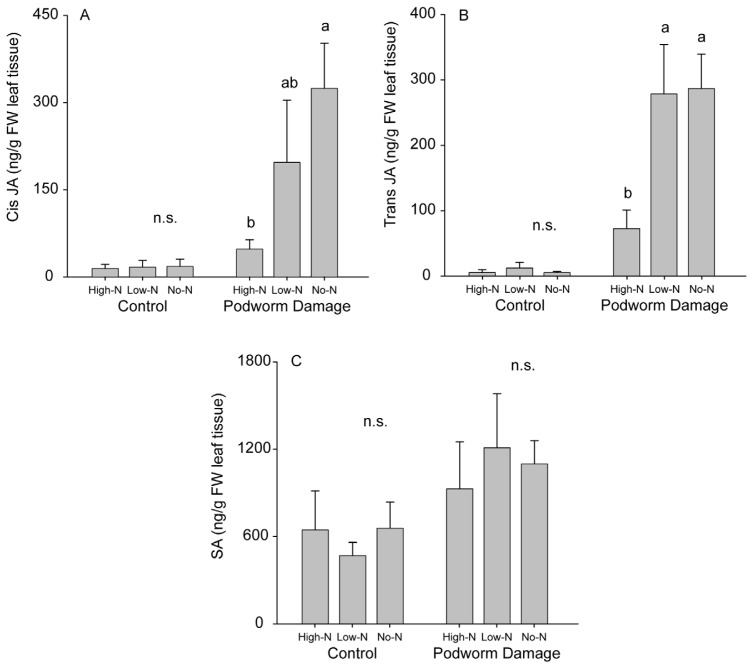
Phytohormone accumulation in undamaged (control) and podworm-damaged soybean with varying dependence on rhizobial N: (**A**) *cis* JA; (**B**) *trans* JA; (**C**) SA. High-N = High N fertilizer treatment, Low-N = Low N fertilizer treatment, and No-N = no fertilizer. Different lowercase letters indicate significant differences for that characteristic, while n.s. indicates no significant difference. Results shown as means ± S.E.

The intensity of rhizobial dependence also influenced plant responses to podworm feeding. Podworm-induced accumulation of JA was affected by the N treatments and resulting variation in rhizobia dependency. After 10 days of feeding damage, *cis* JA was lower in the High-N plants compared to No-N plants (with an intermediate amount in Low-N plants) (Figure 3A; *F*_2,15_ = 6.39; *p* = 0.012), and trans JA was lower in the High-N plants as compared to Low-N and No-N plants (Figure 3B; *F*_2,15_ = 6.53; *p* = 0.011). However, levels of SA were similar across nutrient treatments (Figure 3C; *F*_2,15_ = 0.55; *p* = 0.588). The percent leaf area damaged by podworm feeding was similar across all nutrient treatments (*F*_2,15_ = 1.70; *p* = 0.222; Means + SE: No-N = 31.0 ± 3.7%, Low-N = 26.0 ± 2.4%, High-N = 23.3 ± 2.8%), and phytohormone levels of undamaged control plants were not affected by nutrient treatment (Figure 3A–C; *cis* JA: *F*_2,11_ = 0.15, *p* = 0.864; *trans* JA: *F*_2,11_ = 0.57; *p* = 0.583; SA: *F*_2,11_ = 0.18; *p* = 0.840).

Chewing herbivores typically induce the accumulation of the defense-signaling compound JA, which regulates defense responses [40]. In soybeans, JA-activated defenses (lipoxygenases and protease inhibitors) are induced by podworm feeding and reduce the growth of subsequent larvae added to previously damaged plants [41]. In our phytohormone experiments, all nutrient treatments incurred similar amounts of damage by podworm larvae, but No-N plants accumulated significantly higher levels of JA than High-N plants. The stronger response to damage in plants primarily dependent on rhizobia for N may reflect a degree of defense priming due to a strong association with the bacteria, as has been reported for other plant-beneficial microbe systems, particularly mycorrhizal fungi and nonpathogenic rhizobacteria [9,42,43]. Priming entails an initial activation of the plant defense system resulting in a more intense or rapid induction of defense responses to subsequent environmental challenges [44,45]. For instance, association with nonpathogenic rhizobacteria enhanced plant resistance against a generalist (but not a specialist) chewing herbivore and intensified the expression of JA-responsive defense genes upon damage [46]. Prior to biotic attack, immune responses to beneficial microbes are typically weak, if detectable at all [43], consistent with the low levels of JA and SA measured in our undamaged plants regardless of the intensity of association with rhizobia.

Higher levels of JA induced in No-N plants might be expected to result in increased resistance against podworm herbivory. For example, Katayama *et al.* [12] found increased levels of phenolics in soybeans as rhizobial association increased. However, larvae in our experiment grew better on plants that accumulated higher levels of herbivore-induced JA. This unexpected pattern has also been found in other beneficial microbe/herbivore interactions [47] and may be explained if improved nutritional quality outweighs the effect of elevated defense responses. Nutritional quality is known to influence the effects of defensive compounds on herbivores: optimally balanced diets can render defensive compounds ineffective while suboptimal diets can intensify their impacts [48]. If plant traits associated with higher levels of dependence on rhizobia for nitrogen (e.g., elevated foliar ureide concentrations) enhance the nutritive value for soybean podworm larvae, then the herbivore may be able to better tolerate higher levels of defensive compounds. Stronger induction of JA-mediated defenses might, however, explain the somewhat surprising preference of podworms for High-N plants in choice assays (despite their reduced performance on these plants).

### 2.3. Soybean-Rhizobia Interactions with a Phloem-Feeding Herbivore

**Figure 4.**
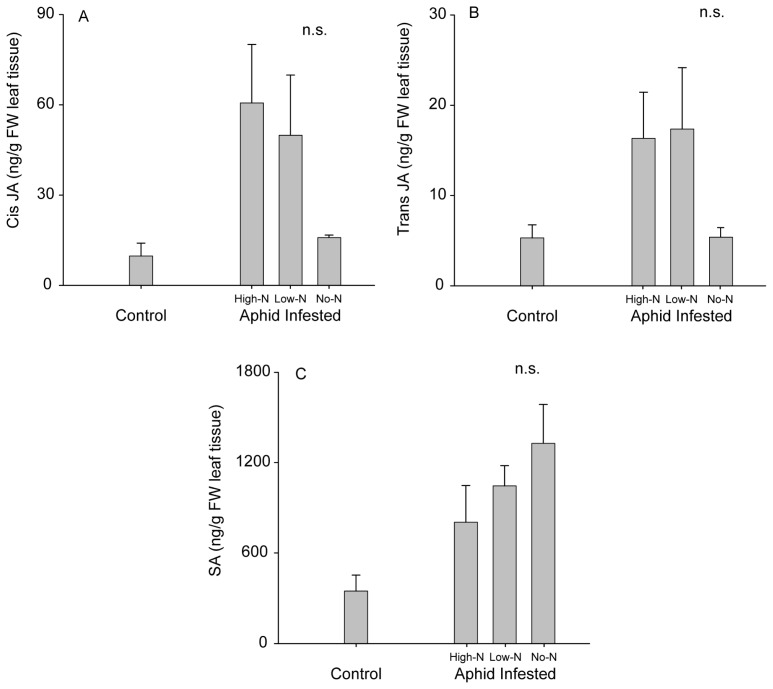
Phytohormone accumulation in undamaged (control) and aphid-infested soybean with varying dependence on rhizobial N: (**A**) *cis* JA; (**B**) *trans* JA; (**C**) SA. High-N = High N fertilizer treatment, Low-N = Low N fertilizer treatment, and No-N = no fertilizer. n.s. indicates no significant difference. Results shown as means ± S.E.

**Figure 5.**
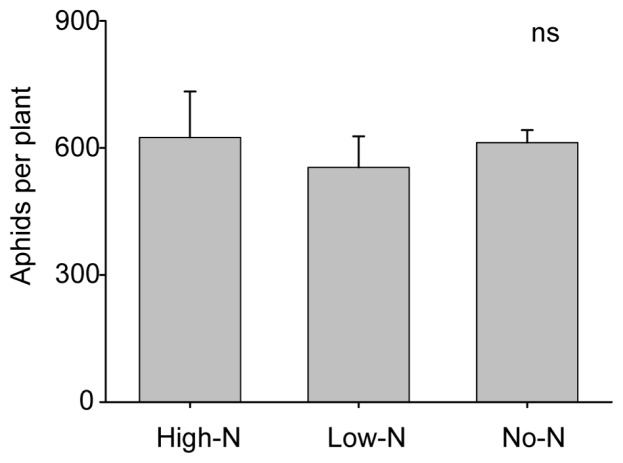
Effects of dependence on rhizobial N by 5-week old soybeans, on the population growth of soybean aphids over 8 days (starting from 15 adults per plant). High-N = High N fertilizer treatment, Low-N = Low N fertilizer treatment, and No-N = no fertilizer. “ns” indicates no significant difference. Results shown as means ± S.E.

In contrast to our results regarding podworm feeding, we found little evidence that plant dependence on rhizobial N influenced interactions with the soybean aphid. Plant nutrient and rhizobial status did not appear to affect population growth of soybean aphids or plant responses to aphids. Although there was a trend towards lower JA levels in No-N plants than High-N and Low-N with aphid feeding, no significant differences were found in aphid-induced JA or SA accumulations among the nutrient treatments (Figure 4A–C; *cis* JA: *F*_2,11_ = 3.77; *p* = 0.065; *trans* JA: *F*_2,11_ = 3.29; *p* = 0.084; SA: *F*_2,11_ = 1.43; *p* = 0.288). Aphid populations did not exhibit differences in growth rates across different nutrient treatments, as there were no significant differences in final populations sizes despite a ~40 fold population increase over the course of the assay (Figure 5; *F*_2,9_ = 0.24; *p* = 0.792), although it remains possible that relatively subtle differences in performance might manifest over longer periods of time.

As phloem feeders, soybean aphids may be less likely to be affected by changes in the proportion of nitrogen present as ureides under different intensities of rhizobial association. Ureides are transported in the xylem and accumulate in the shoots where they are degraded by enzymes before entering the phloem [49]. Consequently, no differences in ureide concentration were reported between phloem of nitrate-fed and rhizobia inoculated soybeans [50]. We also found no significant differences in the levels of JA and SA that accumulated in aphid-infested plants with different levels of rhizobial dependence—though there was a trend toward lower JA induction in No-N plants.

The lack of observed impacts of rhizobia association on plant-aphid interactions in this study (in which all plants were inoculated using one rhizobia inoculant source) bears comparison to a previous field study comparing different strains of rhizobia. In that study we found that soybean plants (cultivar Garst 2918) associating with naturally occurring rhizobia strains hosted significantly lower aphid populations compared to plants inoculated with commercial rhizobia or given N fertilizer [3], highlighting the possibility that different rhizobia strains could impart other effects on aphid-soybean interactions.

Furthermore, as soybean genotype strongly influences the level of resistance against soybean aphid, the use of other soybean cultivars may reveal further information about the effects of rhizobial intensity on this specialized herbivore. The cultivar we used in this study, Williams 82, is classified as susceptible to soybean aphid, and aphid colonies exhibit rapid growth on this cultivar compared to more resistant soybean genotypes [51]. However, even high numbers of aphids do not typically kill Williams 82 plants, in contrast to other susceptible soybean cultivars [51], suggesting a high level of aphid tolerance. While the hormonal responses of resistant and susceptible plants to aphids have been previously examined [25], much less is known about responses of tolerant plants to aphid feeding.

## 3. Experimental Section

### 3.1. Plant Growth

Soybean seeds (Williams 82) were rinsed with a 10% sodium hypochlorite solution followed by copious amounts of water, then placed on wet perlite to germinate for 2 to 3 days. Sprouted seeds were planted in an autoclaved sand/perlite mixture in plastic pots (12 mm diameter by 12 mm tall; Nursery Supplies, Inc., Fairless Hills, PA, USA) lined with coffee filters. All plants were inoculated with rhizobia by adding 1 mL of a *Bradyrhizobium* mixture (HiStick Liquid, Becker Underwood, Ames, IA, USA) to the hole where the seed was planted. Plants were watered with one of three diluted, modified Hoagland’s nutrient solutions with varying amounts of nitrogen (0, 10, and 20 mM) provided as calcium nitrate and ammonium nitrate [30]. Five-week old plants were used for the experiments and were maintained in growth chambers on a 16:8 light:dark cycle.

### 3.2. Insects

Soybean podworm eggs were obtained from the USDA/ARS facility in Tifton, GA, USA, and larvae were reared on an artificial casein-based diet. For the detached leaf relative-growth-rate and preference experiments, second-instar larvae showing signs of molting were removed from diet the day before the experiment and placed in empty diet cups with sufficient moisture. By the day of the experiment, all larvae had freshly molted to the third-instar and had empty guts. For the growth rate experiment using intact plants, caterpillars were taken as freshly hatched neonates and placed on plants.

Soybean aphids were obtained from natural populations at the Russell E. Larson Agricultural Research Center in Rock Springs, PA, USA, and maintained in a growth chamber on soybean plants, which were replaced weekly.

### 3.3. Plant Assessments

Plants were evaluated for the effects of soil nitrogen on plant growth, rhizobial inoculation, and foliar nitrogen forms. Five-week-old plants (*n* = 6) from each of the three nutrient treatments were dried at 50 °C for 72 h, weighed, and ground for nitrogen assessments. Total percent foliar nitrogen was determined by the combustion method (PSU Agricultural Analytical Services Laboratory, University Park, PA, USA). Ureide (allantoin) content was determined colormetrically as the phenylhydrazone derivative of glyoxylate [52]. Nodules were removed from the roots and dried to assess nodulation intensity via dry weight.

### 3.4. Herbivory by Soybean Podworm

The effect of nitrogen status and rhizobial dependency of soybean on soybean podworm growth was assessed in two separate assays. First, the growth rates of third-instar larvae on soybean leaves from plants of the different nutrient treatments were determined in no-choice trials. Young leaves that were near fully expanded were detached and placed in plastic diet cups (30 mL, Solo Cup Co., Urbana, IL, USA) containing 1.5% agar to retain moisture. Freshly molted larvae were weighed before being placed in cups, allowed to feed for 48 h in incubators (14-h photophase, 25 °C, 60% RH), and then weighed again. Between 18 and 21 larvae were tested on individual leaves from plants of each nutrient treatment. Relative growth rate (RGR) represents the difference in initial and final weights of each individual divided by the number of days of feeding weighted by the initial weight [53]. The amount of leaf area consumed was determined by digitally scanning the remaining material (SigmaScan Pro 5, Systat Software, Inc., San Jose, CA, USA).

The second assay—intended to evaluate podworm growth in a more biologically realistic way—was conducted by adding freshly hatched larvae to intact plants and allowing them to feed for eight days in a growth chamber set to a 16-hour photophase. Five freshly hatched neonates were enclosed on the first fully expanded leaf of each plant (five plants per nutrient treatment), using a plastic clip cage constructed from a modified plastic tube with mesh ends that permitted air flow through the leaf. The clip cage and enclosed larvae were moved to fresh leaflets daily until the fourth day, at which point the cages were removed and the plants were covered with mesh bags, allowing larvae to feed freely over the entire plant. After a total of eight days of feeding, the larvae were removed and weighed. A few larvae escaped from the mesh bags over the course of the experiment, resulting in a total of 18 to 20 larvae counted for each nutrient treatment. The initial weights of neonates were estimated as zero because individuals weighed less than allowed by the precision of the balance.

The ability of podworm larvae to distinguish between plants receiving different ratios of nitrogen from rhizobia was examined through preference tests conducted in 6-well Multiwell™ tissue culture plates (Becton Dickinson and Company, Franklin Lakes, NJ, USA), in which leaf disks cut with a #8 cork borer (approx. 100 mm^2^) were pinned to foam covered with moist filter paper. Each arena contained three disks, one each from similar-aged (young, near fully expanded) leaves of plants with the three nutrient treatments (*n* = 30). One larva was allowed to feed in each arena for 5.5 h. Over this time period, larvae typically tasted each disk, and by the end of the assay had typically consumed about half of one disk. At the end of the trial, larvae were removed and the leaf disks digitally scanned for area analysis via SigmaScan Pro 5 (Systat Software, Inc., San Jose, CA, USA). Data was analyzed by comparing the amount consumed from each disk as a proportion of total consumption in each trial, with individual larvae used as blocks for analysis of variance.

In order to assess the effects of rhizobial dependency and nitrogen status on podworm-damage-induced changes in phytohormone concentrations (JA and SA), five neonate larvae were added to clip cages on young leaves of six plants per nutrient treatment and allowed to feed for four days, after which the plants were enclosed in mesh bags and larvae were allowed to roam within the bags. Plants were kept in a growth chamber with a 16-hour photophase and watered as needed with the appropriate nutrient solutions. During the experiment, one plant of each of the No-N and Low-N treatments were inadvertently damaged and discarded from sampling, resulting in a sample size of five for these treatments. Undamaged control plants (*n* = 4) received clip cages and mesh bags without larvae. After a total of 10 days of feeding, one near fully expanded leaf from each plant was detached at the petiole and flash frozen in liquid nitrogen, and percent leaf area consumed digitally assessed using the method above. Samples were stored at −80 °C prior to phytohormone analyses (see below).

### 3.5. Herbivory by Soybean Aphid

The reproductive potential of aphids on plants of each nutrient treatment (*n* = 4) was evaluated by placing 15 adult apterous females of similar size on the upper side of the youngest fully expanded leaf and contained by a clip cage. Plants were maintained in a growth chamber with a 16-hour photophase. After four days, clip cages were removed from the leaves, and each plant was covered in a mesh bag to allow aphids to move over the entire plant. After 8 days, final aphid populations were counted.

During this experiment, control plants were also placed in the chambers and received the clip cage and mesh bag, but no aphids, to evaluate phytohormone levels after eight days of aphid infestation. After eight days, one near fully expanded leaflet was detached from each plant at the petiole and flash frozen in liquid nitrogen, and samples were stored at −80 °C until phytohormone analyses (see below). Some control plants were discarded after being fed upon by escaped aphids or inadvertently damaged, resulting in less than the minimum three plants per nutrient treatment required for statistical analyses. As our overall goal was to compare the phytohormone levels of infested plants from the different nutrient treatments (*n* = 4), the remaining undamaged control plants were combined into one overall control group for informal comparisons with the infested plants.

### 3.6. Phytohormone Analyses

Phytohormones were analyzed from leaf tissue with a method developed by Schmelz *et al.* [54] and modified by Tooker and De Moraes [55]. Briefly, an acidified 1-propanol extraction solution and phytohormone standards (2H6-SA and dihydrojasmonic acid) were added to leaf tissue (~100 mg FW), followed by methylene chloride. The carboxylic acids extracted were derivatized to methyl esters with trimethylsilyldiazomethane, and the methyl esters were collected by vapor phase extraction onto adsorbent polymer filters (SuperQ, Alltech Associates, Deerfield, IL, USA). The filters were eluted with dichloromethane and samples were analyzed by GC-MS (Agilent model 5890 gas chromatograph, Wilmington, DE, USA), with isobutene chemical ionization in the selected-ion mode. Hormones were quantified relative to the internal standards and retention times confirmed by standard curves of pure compounds.

### 3.7. Statistics

Main effects of nutrient treatments from each experiment were tested with one-way ANOVAs and all pairwise treatment mean comparisons were made using Tukey’s HSD test at *p* < 0.05 (using Statistix 8.0; Analytical Software, Tallahassee, FL, USA). Analyses of phytohormone concentrations necessitated a natural log transformation to satisfy the assumptions of normality, except for the SA levels of aphid-infested plants.

## 4. Conclusions

Our results indicate that the intensity of the mutualistic association between soybean and bradyrhizobia—as modulated by soil conditions—can influence interactions between host plants and herbivores, though effects may vary for different classes of herbivores. Specifically, we found that variation in plant dependency on rhizobial N influenced both the feeding preferences and performance of the soybean podworm (a polyphagous foliage feeder), as well as podworm-induced accumulation of the phytohomone JA, which is known to mediate plant defense responses against chewing herbivores. In contrast, we did not observe significant treatment effects on the performance of the soybean aphid (a specialized phloem feeder). This pattern of effects may be explained by rhizobia associated changes in plant nutritional quality, interactions between rhizobia- and pathogen-induced signaling pathways, or quite likely a combination of these effects. Continued elucidation of the effects of rhizobia association on plant-insect interactions will improve our understanding of herbivore dynamics in natural and agricultural legume communities and provide more general insights into the interaction of simultaneous plant associations with multiple organisms.
